# Work demands and physical activity in hospital employees with different degrees of musculoskeletal pain: descriptive data from the STUNTH study, Norway

**DOI:** 10.1186/s12891-026-09972-x

**Published:** 2026-05-21

**Authors:** Roar Munkeby Fenne, Sigmund Østgård Gismervik, Lene Aasdahl, Tom Ivar Lund Nilsen, Eivind Schjelderup Skarpsno, Signe Lohmann-Lafrenz, Ellen Marie Bardal

**Affiliations:** 1 Department of Neuromedicine and Movement Science, Norwegian University of Science and Technology (NTNU), Trondheim, Norway; 2 Department of Public Health and Nursing, Norwegian University of Science and Technology (NTNU), Trondheim, Norway; 3 Unicare Helsefort Rehabilitation Center, Rissa, Norway; 4 Clinic of Emergency Medicine and Prehospital Care, St. Olav’s Hospital, Trondheim University Hospital, Trondheim, Norway; 5 Clinic of Rehabilitation, St Olavs Hospital HF, Trondheim University Hospital, Trondheim, Norway

**Keywords:** Musculoskeletal pain, Work demands, Occupational physical activity, Hospital employees

## Abstract

**Background:**

Healthcare employees are frequently exposed to several risk factors for musculoskeletal pain at work, including high physical demands, psychological stressors, and high levels of occupational physical activity. We aimed to describe self-perceived work demands and device-measured physical activity at work among hospital employees across different occupations and clinical settings experiencing no pain, short-term pain, or long-lasting pain.

**Methods:**

We used cross-sectional data on 1,413 hospital employees who participated in the first wave of the STUNTH (The Study of New Technology and Health among Hospital employees) cohort study. Physical activity was captured by two Axivity AX3 triaxial accelerometers placed on the thigh and lower back during up to seven consecutive days. Self-perceived work demands at the most demanding shift during the same period were assessed by NASA-Task Load Index (overall score and physical and mental demands subscales), ranging from 0 “Very low demands” to 100 “Very high demands”. Musculoskeletal pain was measured by the Norwegian Pain Society Minimum Questionnaire, categorized by pain duration: no pain, short-term (during the last 7 days), or long-lasting (> 3 months). Quantile median regression models adjusted for age and sex were used to estimate work demands and physical activity according to different characteristics of musculoskeletal pain.

**Results:**

The overall prevalence of musculoskeletal pain was 75.7%, where 31.6% reported short-term pain during the previous seven days, and 44.1% reported having pain lasting > 3 months. Both perceived overall work demands and perceived physical work demands were higher for those reporting long-lasting musculoskeletal pain compared to those without pain, with a median difference of 5.8 (95% CI: 3.35 to 8.21) and 10.3 (95% CI: 1.51 to 19.02), respectively. There were no clear differences in mental demands or in device-measured physical activity between employees with or without musculoskeletal pain.

**Conclusion:**

Employees with long-lasting musculoskeletal pain reported higher perceived overall work demands and perceived physical demands, but no significant differences in proportion of time spent in device-measured physical activity types compared to employees without musculoskeletal pain.

**Supplementary Information:**

The online version contains supplementary material available at 10.1186/s12891-026-09972-x.

## Introduction

Musculoskeletal pain (MSP) is a leading cause of long-term sickness absence worldwide [1], and accounted for 33% of all sick leave in Norway in 2023 [2]. MSP has been reported as the most common self-reported work-related health problem among employees in the health care sector [3–5]. A review of musculoskeletal health among nurses showed that approximately 50% experience low-back pain every year [4], while a Swiss study among a broad range of healthcare professionals working in a hospital setting reported that almost 25% had severe- to very severe MSP [6]. Given the high prevalence of MSP in the working population in general [1], the majority will experience MSP to some extent during their working life. While shorter episodes of MSP are common and are most often self-limiting [7, 8], these episodes may affect both workability and well-being [9] and may also develop into long-lasting pain conditions. In light of the expected shortage of healthcare personnel in the next decades [10, 11] it is increasingly important to prevent sickness absence and early retirement due to MSP in order to secure a sustainable healthcare system. Employees in the health care sector are frequently exposed to several acknowledged risk factors for MSP, including high physical and psychosocial work demands [12–15] and high levels of physical activity and prolonged standing at work [16–19], when compared to other sectors and occupations [13, 17]. However, less is known about how work demands are perceived by employees with and without MSP, and whether these demands are consistent with objective measures of occupational physical activity. Experiencing pain at work may alter the perception of work demands [20], and affect work ability and performance [21]. Employees experiencing pain might therefore perceive work tasks as more demanding, despite having the same objective workload as their co-workers without pain. Acute stress reactions to high physical and psychological demands impair recovery processes, which may increase the risks of chronic impairments like prolonged fatigue and long-term musculoskeletal pain [22]. Understanding how MSP affects perceived work demands and occupational physical activity among healthcare workers is important for designing targeted interventions and work environments. These measures can reduce the impact of working with MSP, preventing progression to more severe pain that may ultimately lead to sickness absence or disability. Thus, the aim of this cross-sectional study of hospital employees from a range of occupational groups and clinical settings was to describe self-perceived work demands and device-measured physical activity at work among hospital employees experiencing no pain, short-term pain, and long-lasting pain.

## Method

### Study population and procedure

**Fig. 1:**
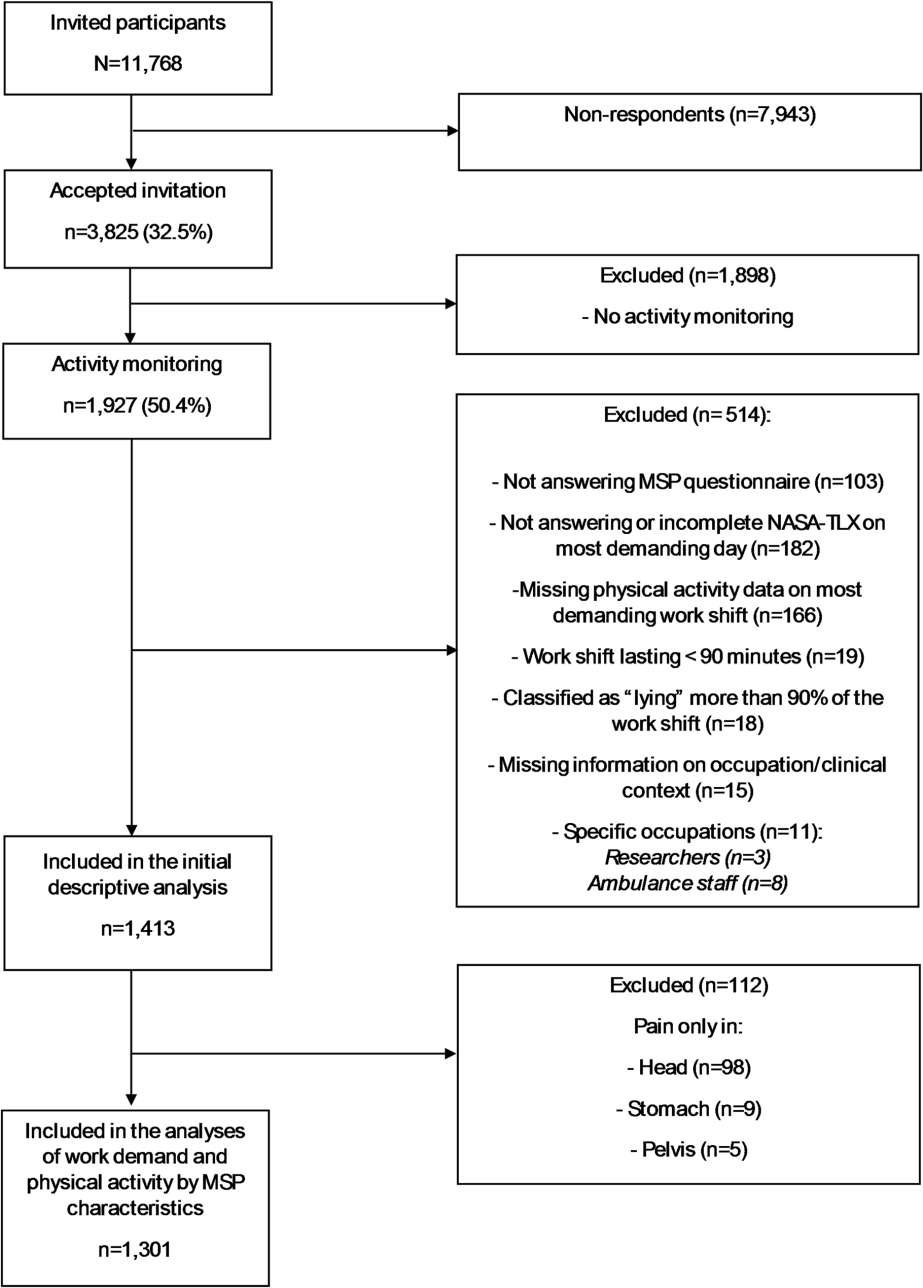
Flowchart of included participants; Abbreviations: MSP = Musculoskeletal pain, NASA-TLX = NASA Task Load Index

This study is based on data from the first data collection of The Study of New Technology and Health among Hospital employees (STUNTH, www.stunth.no), which was conducted between October 2021 to May 2022. The STUNTH-study is an open cohort study of hospital employees in central Norway. All employees at the St. Olavs Hospital HF in Trondheim, Norway, (11,768) were invited to participate. In total, 3,825 (32,5%) accepted the invitation. The data collection included three steps: (1) questionnaires regarding work-life and sociodemographic factors, (2) seven days of objective measurements of physical activity, registration of working hours, and rating of perceived work demands, and (3) questionaries regarding health, sleep and lifestyle factors the week following activity monitoring. For the purpose of the present study, we excluded participants if they on their most demanding work shift had (1) missing physical activity data, (2) incomplete rating of work demands, (3) registrations of lying down more than 90% of the shift or (4) had a shift duration < 90 min. Participants were also excluded if they belonged to occupational groups with less than 15 employees, as stated in the ethical approval. In total, 1,413 participants were available for statistical analysis. A flowchart of the selection procedures is shown in Fig. 1. The present study follows the STROBE guidelines for reporting [23].

### Data collection and analysis

#### Device-measured physical activity

Physical activity was assessed using two Axivity AX3 triaxial accelerometers (Axivity, Ltd., Newcastle, United Kingdom) for seven consecutive days. In addition, the participants registered their working hours in a diary, to be able to separate between physical activity at work and during leisure time. The accelerometers were attached to the skin centrally on the right thigh approximately 10 cm above the upper border of the patella and on the lower back at the level of the third lumbar segment using an adhesive film (Opsite Flexifix; Smith and Nephew) over and under the accelerometers.

Acceleration data was sampled at 50 Hz, with ±8G following the protocol described in Bach et al. 2022 [24]. The raw acceleration data from the two sensors were synchronized and segmented into 5 s windows before using an eXtreme Gradient Boosting (XGBoost) machine learning model to classify activities into lying, sitting, standing, walking, stairs, running and cycling [24]. Time spent in the different physical activities during work was estimated based on time series data of classified activities and the work diary and presented as percentage of total work time. Time spent walking, running and cycling was summarized as “moving”. To describe total time spent in active behavior during work, time spent “moving” and standing was summarized as “time on feet”.

#### Work demands

After seven days of physical activity monitoring, participants rated their perceived work demands based on what they considered their most demanding shift during the week. Work demands were assessed using a translated and modified version of the NASA Task Load Index (NASA-TLX) [25]. The NASA-TLX measures workload using visual analogue scales (0-100) for six sub-dimensions of workload; mental demand, physical demand, temporal, effort, performance, and frustration [25], with 0 indicating low demands and 100 indicating very high demands. An overall work demands score was calculated by averaging the six unweighted NASA-TLX dimensions. While the NASA-TLX originally includes a weighting evaluation of all pairs of sub-dimensions by the participants prior to data collection, the use of unweighted raw NASA-TLX (RTLX) scores are increasingly used in the literature as it simplifies the data collection process, while still showing a strong correlation to the weighted NASA-TLX score [26, 27].

#### Musculoskeletal pain

MSP during the last seven days was assessed using questions from the Norwegian Pain Society Minimum Questionnaire [28], which are translated and validated questions from the Brief Pain Inventory questionnaire [29]. The location of MSP was assessed using a pain map with 25 locations. Participants were defined as having MSP if they registered pain in one or more body locations. Pain regions were reported separately for the left and right side of the body but collapsed into a single category in the analyses, e.g., pain in the left and/or right knee was combined into the category “knees”. Participants that only reported pain in the head (*n* = 98), stomach (*n* = 9), or pelvis region (*n* = 5) were omitted in the analyses related to MSP due to potential non-MSP causes. Intensity of average pain during the last seven days was assessed with a single question, regardless of body location, on a 0–10 numerical scale, with 0 indicating “no pain” and 10 indicating “worst imaginable pain”. We analyzed pain intensity as both a continuous variable, and a categorical variable, by grouping scores into clinically relevant meaningful categories based on established thresholds [30, 31]: no pain (0), mild pain (1-3), moderate pain (4-6), severe pain (7-10), with pain status defined irrespective of body region. Lastly, long-lasting MSP was assessed with the question “Have you been bothered by pain in muscles and joints continuously for at least 3 months during the past year?” (Yes/No). We categorized MSP into three groups based on the questionnaire responses: (1) no pain; participants that answered “no pain last week”, (2) short-term pain; participants who reported pain in at least one body location the past seven days and answered “no” to pain lasting 3 months, and (3) long-lasting pain; participants who reported pain the last seven days and answered “yes” to pain lasting 3 months.

#### Other variables

Information about age (years), sex (male/female), type of employment (permanent/temporary and employment fraction), occupation and clinical context was collected from the administration system at the hospital. Smaller occupational groups, with similar educational backgrounds or work tasks were merged into one group, e.g., physiotherapists and occupational therapists were merged into “Therapists”.

#### Statistical analysis

Descriptive statistics are given as frequencies with percentages for categorical variables and as means with standard deviations (SDs) or medians with interquartile range (IQR) for continuous variables. Our study population consists of a diverse range of occupations working in different clinical settings, hence descriptive statistics of the outcome variables are presented within occupations and clinical contexts in addition to results for the overall study population.

Due to the skewed distribution of all outcome variables, we used multiple quantile regression models to estimate median differences in work demand scores, and in % of time spent in physical activity behaviors (i.e., time on feet, standing, moving, sitting) between different categories of MSP (i.e., no pain, short-term pain, and long-lasting pain*)*, and pain intensity. The main analyses included the overall NASA-TLX score and the mental and physical demand subscales as they are established risk factors for musculoskeletal pain [14, 15]. All six NASA-TLX subscales are described descriptively in Additional file 3, and regression analyses for all subscales are presented in Additional file 4. Quantile regression models the linear relationships with specific quantiles (e.g., median). When modeling the median (i.e. 0.5 quantile), the coefficients represent the change in the conditional median of the outcome for a one-unit change in the predictor, holding the other variables constant. We used “no pain” as the reference when comparing differences between MSP status, and participants scoring 0 on pain intensity scale as reference for the pain intensity group comparisons. All associations were adjusted for age (continuous) and sex (female, male). Due to the descriptive nature of this study, adjustment for other factors was not considered [32]. We assessed the precision of the estimates by 95% confidence intervals (CIs). All analyses were performed using Stata 19 (StataCorp. 2025. Stata Statistical Software: Release 19 College Station, TX: StataCorp LLC).

### Ethics

The study protocol was approved by the Regional Committee for Medical and Health Research Ethics in Central Norway (Ref. 228249). All participants gave their written informed consent before entering the study. The study was conducted in accordance with the ethical principles of the Declaration of Helsinki [33].

## Results

### Descriptives of study population

The included participants had a mean age of 42.6 years (SD 12.4). Most participants were female (83.9%), and a majority (79.1%) held a permanent position and were full time employed (72.5%). The participants were grouped into 12 occupational groups, working within 9 different clinical contexts. Nurses (22.7%) and specialist nurses (19.6%) were the two largest occupational groups, followed by laboratory technicians (10.0%) and senior doctors (8.2%). See Additional file 1 for a complete description of the participants.

MSP, self-perceived work demands, and device-measured physical activity of participants within each occupational group and clinical context are described in Tables 1 and 2. The overall prevalence of MSP was 75.7% (*n* = 985), of which 31.6% reported short-term MSP and 44.1% reported long-lasting MSP (Table 1). The median average pain intensity during the week of participation was 3.0 (IQR 2.0–4.0). The overall prevalence of MSP varied across occupational groups, with laboratory technicians, and facility- and support staff having the highest prevalence (82.6% and 82.4% respectively). Regarding long-lasting pain, the highest prevalence was found among specialist nurses (52.2%) and administrative personnel (50%). Within clinical contexts (Table 2), employees working in laboratory medicine (50%) reported the highest prevalence of long-lasting MSP. See Additional file 2 for distribution of pain locations.

**Table 1:** Musculoskeletal pain, median work demands score and median physical activity distribution within different occupations

	Musculoskeletal Pain^a)^ (Prevalence and pain intensity)			Work Demands^b)^ (Measured by NASA-TLX scores from 0-100)			Physical Activity (Percent of worktime spent in different categories)			
	Short-term (%)	Long-lasting (%)	Pain intensity (NRS, 0–10) Median (IQR)	Overall, Median (IQR)	Mental, Median (IQR)	Physical, Median (IQR)	Total time on feet^c)^, % (IQR)	Standing, % (IQR)	Moving^d)^, % (IQR)	Sitting, % (IQR)
Total^a)^ (*n* = 1,413)	31.6	44.1	3 (2–4)	55.5 (45.2–64.9)	72.6 (56.3–82.8)	38.9 (13.7–65.3)	46.0 (28.3–62.1)	36.1 (21.7–51.1)	7.6 (5.0–11.3)	50.6 (35.4–69.4)
Nurses (*n* = 321)	35.5	39.7	3 (3–4)	58.5 (44.2–67.0)	70.5 (54.0–80.0)	52.6 (27.4–72.1)	56.9 (44.1–66.0)	45.9 (33.7–55,6)	8.9 (6.4–12.4)	41.4 (32.3–53.3)
Specialist nurses (*n* = 277)	27.1	52.2	3 (3–4)	57.1 (46.0–66.4)	74.2 (58.6–82.8)	50.5 (21.1–70.7)	55.2 (36.6–67.2)	45.2 (28.7–56.5)	8.8 (5.6–12.2)	42.4 (31.6–59.1)
Laboratory technician (*n* = 143)	37.9	44.7	3 (2–4)	51.1 (43.4–60.0)	72.6 (56.3–79.5)	31.6 (13.6–56.3)	45.4 (33.3–58.8)	37.1 (26.7–49.0)	7.3 (5.2–9.8)	53.6 (40.7–66.0)
Senior doctors^e)^ (*n* = 115)	26.2	39.3	3 (2–4)	55.1 (47.1–60.7)	77.9 (65.3–86.9)	22.1 (10.0–45.3)	28.9 (22.6–43.9)	23.5 (15.3–35.6)	6.4 (4.5–9.0)	67.2 (48.6–75.7)
Administrative personnel (*n* = 103)	30.0	50.0	4 (3–5)	50.8 (42.2–58.4)	72.1 (60.0–80.8)	10.1 (4.0–28.8)	22.8 (13.9–34.1)	17.2 (10.0–30.9)	4.4 (2.2–7.4)	75.8 (60.9– 85.9)
Management (*n* = 96)	18.2	46.6	3 (2–4)	59.2 (49.8–65.5)	78.4 (69.8–87.9)	15.3 (7.1–37.6)	32.9 (20.4–45.4)	23.9 (14.4–37.2)	6.2 (4.8–9.2)	65.9 (51.1–78.1)
Other patient related^f)^ (*n* = 88)	31.3	48.7	4 (3–5)	53.2 (42.1–64.3)	74.5 (55.5–84.2)	21.3 (7.9–61.6)	36.3 (21.1–57.1)	27.6 (14.8–47.5)	6.5 (4.6–9.8)	61.5 (40.4–78.0)
Facility and support staff (*n* = 86)	35.3	47.1	4 (3–7)	57.7 (44.0–66.7)	51.3 (32.3–74.2)	63.9 (48.9–80.0)	70.0 (46.6–80.0)	46.4 (31.5–60.0)	16.7 (9.3–23.1)	28.7 (18.3–50.0)
Imaging technicians (*n* = 58)	32.1	44.6	3 (2–4)	58.2 (51.0–71.0)	69.2 (54.5–80.0)	64.7 (46.3–78.4)	50.8 (42.1–58.6)	39.1 (27.9–50.9)	8.2 (6.2–12.0)	47.2 (33.3–57.8)
Therapists^g)^ (*n* = 48)	28.9	28.9	3 (2–4)	53.9 (45.4–64.8)	73.4 (59.5–87.9)	30.8 (13.9–64.1)	35.0 (28.5–48.5)	28.1 (18.7–37.8)	7.2 (5.6–12.2)	65.0 (51.5–69.8)
Junior doctors^e)^ (*n* = 40)	48.6	17.1	2 (2–3)	51.3 (41.8–63.3)	70.8 (52.6–84.2)	26.6 (9.8–44.5)	37.2 (28.2–46.0)	30.1 (23.2–40.9)	5.6 (3.7–6.9)	57.8 (44.6–70.4)
Psychologists (*n* = 38)	40.0	40.0	3 (3–4.5)	52.9 (45.1–60.4)	81.7 (70.0–88.9)	5.2 (2.6–17.4)	20.5 (14.2–27.9)	14.1 (8.7–20.4)	4.1 (2.8–6.5)	79.5 (72.1–85.8)

*Abbreviations*: *NRS *Numerical Rating Scale, *IQR *interquartile range

a) MSP calculated after excluding those who reported pain in only Head, Stomach or Pelvis (n=112). Total N=1,301

b) NASA-TLX scores from 0-100, with 0 indicating very little demand and 100 indicating very high demand

c) Time on feet = Standing, walking, stairs, cycling, running

d) Moving = Walking, running, cycling, stairs

e) In the Norwegian context, senior doctors have finished their specialization (consultants) and junior doctors are residents in different stages of training

f) Including medical secretaries, healthcare assistants, social workers and pedagogues

g) Including physiotherapists and occupational therapists

**Table 2:** Musculoskeletal pain, median work demands score and median physical activity distribution within different clinical settings

	Musculoskeletal Pain^a)^ (Prevalence and pain intensity)			Work Demands^b)^ (Measured by NASA-TLX scores from 0-100)			Physical Activity (Percent of worktime spent in different categories			
	Short-term (%)	Long-lasting (%)	Pain intensity (NRS, 0–10) Median (IQR)	Overall, Median (IQR)	Mental, Median (IQR)	Physical, Median (IQR)	Total time on feet^c)^, % (IQR)	Standing, % (IQR)	Moving^d)^, % (IQR)	Sitting, % (IQR)
Internal medicine (*n* = 349)	32.6	40.6	3 (3–4)	57.4 (47.5–65.3)	72.7 (56.3–81.8)	39.4 (15.8–65.8)	47.3 (28.8–62.2)	36.2 (22.3–51.0)	8.0 (5.2–11.3)	50.0 (36.1–39.6)
Surgery (*n* = 222)	33.5	43.2	3 (3–4)	57.3 (46.0–67.3)	71.9 (57.6–81.6)	52.8 (22.1–71.6)	53.9 (38.6–68.0)	44.7 (30.6–57.3)	8.0 (5.8–11.7)	41.4 (30.5–57.7)
Anesthesiology (*n* = 191)	28.8	46.0	3 (3–4)	58.2 (46.1–68.4)	76.8 (64.2–87.4)	49.5 (17.4–70.7)	56.2 (36.8–68.4)	47.2 (31.1–59.3)	7.5 (4.5–11.5)	40.7 (29.7–56.3)
Laboratory medicine ^e)^ (*n* = 156)	34.5	50.0	3 (2–4)	51.2 (43.2–60.4)	71.8 (55.1–82.0)	28.1 (12.9–55.8)	44.7 (29.1–56.7)	35.7 (24.5–48.1)	7.1 (5.1–9.6)	54.8 (42.6–70.4)
Psychiatry (*n* = 143)	29.3	45.9	3 (2–4)	52.5 (43.2–59.6)	73.7 (61.1–84.7)	15.3 (5.3–30.5)	27.4 (18.7–37.5)	21.2 (12.3–28.2)	6.2 (3.8–9.1)	71.1 (60.7–79.6)
Other contexts^f)^ (*n* = 108)	27.2	48.5	3 (2–4)	54.3 (42.2–64.9)	71.7 (52.8–85.4)	40.0 (18.6–66.0)	47.3 (27.8–59.6)	37.2 (21.2–46.9)	8.0 (5.4–11.9)	50.1 (38.5–67.1)
Maintenance (*n* = 94)	38.0	43.5	4 (3–6)	53.3 (42.9–65.3)	63.9 (41.1–78.8)	56.8 (16.3–74.7)	58.0 (31.7–77.9)	38.9 (23.5–55.2)	11.6 (6.3–19.8)	40.1 (20.7–63.3)
Radiology (*n* = 80)	31.2	45.4	3 (2–4)	56.4 (47.8–68.4)	72.7 (54.9–87.4)	38.9 (7.7–69.3)	38.3 (22.8–52.5)	31.0 (18.3–44.9)	6.7 (3.4–8.9)	58.4 (44.4–73.2)
Prevention and rehabilitation (*n* = 36)	30.3	30.3	3 (2–3.5)	55.1 (48.9–66.1)	73.7 (58.3–87.9)	33.7 (14.1–68.4)	40.7 (30.9–51.2)	29.7 (22.8–44.1)	7.1 (5.7–10.4)	59.4 (48.8–67.6)
Central administration and management (*n* = 34)	18.8	42.4	4 (3–4)	53.7 (46.0–62.6)	74.7 (65.8–77.9)	9.1 (4.2–51.6)	28.8 (19.0–41.8)	20.8 (11.4–32.0)	6.6 (3.4–10.6)	69.5 (56.6–80.0)

*Abbreviations*: *NRS *Numerical Rating Scale, 0-10; *IQR *interquartile range

a) MSP calculated after excluding those who reported pain in only Head, Stomach or Pelvis (n=112). Total N=1,301

b) NASA-TLX scores from 0-100, with 0 indicating very little demand and 100 indicating very high demand

c) Time on feet = Standing, walking, stairs, cycling, running

d) Moving = Walking, running, cycling, stairs

e) Including laboratory technicians and neurophysiology technicians

f) Including acute medicine, gynecology & obstetrics, otorhinolaryngology

### Perceived work demands

The median overall perceived work demands score was 55.5 (IQR 45.2–64.9) (Table 1), ranging from 50.8 (IQR 42.2–58.4) among administrative personnel to 59.2 (IQR 49.8–65.5) among management personnel. Psychologists reported the highest mental demands (median: 81.7; IQR: 70.0–88.9), whereas facility and support staff reported the highest physical demands (median: 63.9; IQR: 48.9–80.0).

Within clinical contexts, the overall work demands score ranged from 51.2 (IQR 43.2–60.4) within laboratory medicine to 58.2 (IQR 46.1–68.4) within anesthesiology (Table 2), which also reported the highest mental demands (median: 76.8; IQR: 64.2–87.4). Physical demands were highest in maintenance (56.8; IQR: 16.3–74.7). Results from all sub-dimensions of work demands by occupational groups are presented in Additional file 3.

### Physical activity at work

Overall, the median time spent on feet (i.e., moving or standing) was 46.0% (IQR 28.3–62.1) of the work shift (Table 1). Within occupational groups, facility and support staff spent most time on feet (70.0%, IQR 46.6–80.0), followed by nurses (56.9%, IQR 44.1–66.0) and specialist nurses (55.2%, IQR 36.6–67.2). Within clinical contexts (Table 2), employees in maintenance (58.0%, IQR 31.7–77.9) and anesthesiology (56.2%, IQR 36.8–68.4) spent most time on feet. Sitting was the most prominent activity overall during the work shift (median: 50.6%, IQR: 35.4–69.4), while standing was the most prevalent time on feet behavior (median: 36.1%, IQR: 21.7–51.1).

### MSP and work demands

Table 3 shows the median and the estimated median difference in overall work demands score and the scores for the two sub-dimensions mental demands and physical demands, within different categories of MSP and pain intensity. There were no clear differences in perceived work demands between participants with short-term MSP and those without pain. Participants with short-term MSP had 8.1 (95% CI: -1.2 to 17.4) higher score in perceived physical demands compared to those without MSP. Those with long-lasting MSP reported 5.8 (95% CI 3.35 to 8.21) higher overall work demand score compared to those not having MSP. Perceived physical demands were also higher among those having long-lasting MSP compared to not having MSP (difference in median: 10.3; 95% CI: 1.51 to 19.0). In terms of pain intensity, a one-point increase in pain intensity score on the NRS (0–10), was associated with a 1.3 (95% CI: 0.6 to 2.0) higher overall work demand score, and a 5.3 (95% CI 2.7 to 7.9) higher physical demand score. Median perceived scores, and IQR, for all sub-dimensions of work demands within different categories of MSP and pain intensity are presented in Additional file 4.

**Table 3:** Difference in median perceived overall, mental, and physical work demands, by musculoskeletal pain characteristics

		Perceived work demands					
		Overall work demand		Mental Demand		Physical demand	
Pain variables	No. of Participants	Median	Median difference^a^ (95% CI)	Median	Median difference^a^ (95% CI)	Median	Median difference^a^ (95% CI)
Musculoskeletal pain							
No	316	51.8	Ref	71.3	Ref	30.3	Ref
Short-term	411	54.3	2.3 (-0.3 to 4.9)	70.5	-0.4 (-3.4 to 2.6)	42.1	8.1 (-1.2 to 17.4)
Long-lasting	574	58.1	5.8 (3.4 to 8.2)	74.2	2.75 (-0.1 to 5.6)	42.6	10.3 (1.5 to 19.0)
Pain Intensity							
Continuous (NRS 0–10)	983	56.6	1.3 (0.6 to 2.0)	72.6	0.1 (-0.7 to 1.0)	42.6	5.3 (2.7 to 7.9)
No pain (NRS 0)	36	51.2	Ref	71.6	Ref	44.7	Ref
Mild Pain (NRS 1–3)	741	56.4	5.3 (-0.5 to 11.0)	72.7	3.6 (-2.7 to 9.9)	40.0	12.3 (-8.9 to 33.5)
Moderate pain (NRS 4–6)	193	58.6	7.5 (1.4 to 13.7)	72.2	1.6 (-5.1 to 8.4)	50.3	25.0 (2.3 to 47.6)
Severe pain (NRS 7–10)	13	60.5	9.4 (-1.5 to 20.4)	71.1	1.1 (-10.9 to 13.1)	53.1	30.2 (-10.0 to 70.4)

*Abbreviations*: *CI *Confidence Interval, *NRS *Numeric Rating Scale

^a^ Quantile regression analysis for median difference. Adjusted for age (continuous) and sex (male, female)

### MSP and physical activity

Table 4 shows the median percentage of time spent being on feet, and time spent in the physical activity types sitting, standing, and moving, within different durations and intensities of MSP. There were no clear differences in the percentage of time spent in different physical activity types for those with short-term or long-lasting MSP compared to those without MSP. The difference in median time spent on feet was 2.2% (95% CI: -2.7 to 7.1) higher among those with short-term MSP, and 3.2% (95% CI: -1.4 to 7.9) among those with chronic MSP. We observed slightly higher levels of time spent on feet with increasing levels of pain intensity. A one-point increase in pain intensity was associated with 1.5% (95% CI: 0.1 to 2.9) more time on feet. Compared to participants with no pain, those with mild pain spent 3.6% (95% CI: -7.7 to 14.9) more time on their feet, those with moderate pain spent 7.8% (95% CI: -4.2 to 19.9) more time on their feet, and personnel with severe pain spent 18.4% (95% CI: -3.0 to 39.8) more time on their feet. However, the confidence intervals were wide and also compatible with no or inverse associations. The amount of time spent moving was slightly higher with increasing levels of pain intensity, where a one-point increase in pain intensity level was associated with 0.3% (95% CI: 0.0 to 0.5) higher time moving. However, on a categorical level the difference in median time spent moving was 2.2% (95% CI: 0.1 to 4.2) higher among those with moderate pain intensity, and 5.4% (95% CI: 1.7 to 9.1) higher among those with severe pain, compared to those that reported 0 in average pain intensity.

**Table 4:** Difference in median percentage of time spent in physical activity types, by musculoskeletal pain characteristics

Percent of time in physical activity types									
		Total time on feet^a)^		Standing		Moving^b)^		Sitting	
Pain variables	*N*	Median	Median difference^c)^ (95% CI)	Median	Median difference^c)^ (95% CI)	Median	Median difference^c)^ (95% CI)	Median	Median difference^c)^ (95% CI)
Musculoskeletal pain									
No	316	44.8	Ref	33.8	Ref	8.0	Ref	52.5	Ref
Short-term	411	48.0	2.2 (-2.7 to 7.1)	37.9	3.2 (-1.1 to 7.6)	7.4	-0.7 (-1.5 to 0.2)	48.6	-3.6 (-8.4 to 1.1)
Long-lasting	574	46.2	3.2 (-1.4 to 7.9)	36.0	2.6 (-1.4 to 6.7)	7.6	-0.3 (-1.2 to 0.5)	50.0	-4.6 (-9.1 to -0.2)
Pain intensity									
Continuous (NRS 0–10)	983	46.6	1.5 (0.1 to 2.9)	28.3	1.0 (-0.3 to 2.2)	7.5	0.3 (0.0 to 0.5)	49.8	-0.8 (-2.3 to 0.7)
No pain (NRS 0)	36	45.3	Ref	38.1	Ref	5.8	Ref	49.9	Ref
Mild Pain (NRS 1–3)	741	45.3	3.6 (-7.7 to 14.9)	36.0	-2.1 (-11.7 to 7.5)	7.5	1.4 (-0.6 to 3.3)	50.6	2.2 (-9.1 to 13.6)
Moderate pain (NRS 4–6)	193	49.8	7.8 (-4.2 to 19.9)	38.0	-0.4 (-10.7 to 9.8)	8.3	2.2 (0.1 to 4.2)	48.7	-0.8 (-12.9 to 11.3)
Severe pain (NRS 7–10)	13	59.3	18.4 (-3.0 to 39.8)	47.3	6.7 (-11.5 to 24.9)	11.5	5.4 (1.7 to 9.1)	40.7	-9.8 (-31.3 to 11.7)

*Abbreviations*: *CI *Confidence Interval, *NRS *Numeric Rating Scale, 0-10

a) Time on feet = Standing, walking, stairs, cycling, running

b) Moving = Walking, stairs, cycling, running

c) Quantile regression analysis for median difference. Adjusted for age (continuous) and sex (male, female)

## Discussion

The present study examined self-perceived work demands and device-measured physical activity at work among hospital employees with and without MSP, across a heterogeneous range of occupations and clinical contexts. Compared to those without pain, participants with long-lasting MSP reported higher perceived overall work demands and physical demands. However, there were no differences in perceived mental demands or time spent in different device-measured physical activity types. This indicates that the greater perceived work demands among employees with long-lasting pain are not necessarily due to increased physical activity levels at work, but rather due to other factors related to working with long-lasting pain not necessarily captured by device-based measurements alone.

### MSP among healthcare employees

The high overall prevalence of 75.7% of participants reporting MSP in the present study is in line with previous studies on employees in the health care sector ranging from 75% − 87.8% [6, 34, 35], which indicates a high burden of MSP among employees in the health care sector. Considering long-lasting pain, the prevalence of 44.1% was somewhat lower than the observed prevalence of 48.8% in a general working population in Norway [36]. However, this difference might be explained by a younger study population in the present study [37]. Furthermore, the observed median pain intensity in our study was quite low (3.0). This study includes employees that attend work and might therefore represent a healthier sample of the total population. When examining specific occupations and clinical contexts, there were considerable variations in the distribution between short-term and long-lasting pain. Junior doctors reported the highest prevalence of short-term pain (48.6%), but the lowest prevalence of long-lasting pain (17.1%), while management and administrative staff showed opposite trends. Our results highlight the importance of considering different prevalences of MSP within different occupations and clinical settings.

### Work demands and MSP

Compared to employees without MSP, we found that for the whole sample of hospital employees, those with long-lasting MSP reported higher perceived overall work demands and physical demands. Due to the cross-sectional design of our study, we are not able to establish the direction of the association, but several plausible interpretations exist. One possibility is that employees with long-lasting MSP are exposed to more physically challenging tasks, such as frequent patient handling, repetitive lifting, or prolonged standing – which are known common risk factors for developing MSP [12, 19]. However, we found no clear differences in device-measured physical activity at work when comparing those with either short-term or long-lasting MSP against those without MSP. Hence, the observed discrepancy between subjective and device-measured physical demands suggests that perceived physical demand is influenced by more than activity volume alone. This might indicate that individuals with MSP continue to perform similar physical tasks despite experiencing pain, possibly due to the nature of their job roles or workplace expectations not allowing otherwise. Experiencing long-lasting MSP may alter the perception of work demands, so that tasks that are often considered manageable could be perceived as more strenuous or stressful [20]. Greater exposure to physical work demands has previously been associated with reduced work ability (i.e., the balance between the demands of the work vs. the individual’s capacity to perform the work) among workers with MSP in physically demanding jobs in general [38] and among healthcare workers [9]. The experience of physical demands may also be influenced by the individual’s physical capacity, where a given task may represent a higher relative workload for someone with lower muscular strength or aerobic fitness [39]. Measures of physical capacity should therefore be included in future studies on work demands among employees with MSP.

We observed no difference in perceived mental demands between employees with or without MSP. One interpretation is that there are other factors than increased mental demands that are associated with working while experiencing MSP. On the other hand, mental demands were scored quite high across all occupations, clinical contexts, and MSP categories, which might indicate that working at a hospital is inherently mentally challenging. This may be due to context specific characteristics such as patient handling and care, hectic schedules etc., which might be exacerbated by understaffing or sick leave.

### Physical activity at work

The distribution of time spent in the various physical activity types are similar to what has been measured among home care workers in Norway [40]. This similarity may reflect a broader trend in physical activity exposures at work within healthcare occupations in Norway. At the same time, the present study adds to this knowledge by providing a more detailed assessment of physical activity at work across a wide large range of healthcare and non-healthcare occupations within a hospital.

We observed a modest linear increase in self-perceived overall work demands, physical work demands, and proportion of time spent moving with increasing levels of pain intensity in the whole sample of hospital employees. Although the differences were relatively small in absolute terms, they may reflect increased task load or compensatory movement behaviors undertaken in response to discomfort. Alternatively, individuals with higher pain intensity may occupy roles that require more physically demanding tasks. Even though the estimated difference between pain intensity groups and physical activity are uncertain due to the small sample of participants, participants with severe pain intensity spent a substantial amount of more time on feet at work (+ 18%) and less time sitting (-9%) compared to those with no pain. Given the limited sample size with severe pain, future studies in larger populations are needed to confirm this association and establish whether excessive time on feet represents a modifiable risk factor for severe pain. Previous studies have linked more time spent sitting at work with a reduction in pain intensity among healthcare workers [41] and blue-collar workers [42, 43]. Introducing bouts of sitting during the workday may be a beneficial and cost-effective measure to reduce the impact of MSP in this group and potentially lower sickness absence and disability. Again, due to the cross-sectional design, these findings should be interpreted cautiously as temporal relations cannot be determined. In addition, the observed associations may be influenced by residual confounding as not all relevant factors were accounted for in this descriptive study. However, exhaustive adjustment for all potential confounders is neither required nor standard practice for descriptive studies where the aim is to generate hypotheses and describe population patterns rather than to infer causality [32]. We therefore limited the adjustment to basic demographic factors (age and sex), consistent with the descriptive scope of this study. Nonetheless, the findings align with the concept that pain and physical work demands may co-occur in a complex manner through multiple pathways.

Insights from descriptive studies such as this present study can identify patterns of health and work-related stressors which can serve as a foundation for future longitudinal hypothesis-driven research and inform policy decisions [32]. Combining subjective and device-based measurement of work demands, incorporating context-specific measures, and examining the compositional relationship between physical activity at work and during leisure time, may offer a more comprehensive understanding of how physical behaviors and lifestyle factors impact musculoskeletal health. The high prevalence of MSP among a heterogenous groups of hospital employees, together with the large variation in work demands and physical activity levels at work described in our study, highlights the complexity of the relation between different work-related risk factors for MSP and the need for future research that accounts for differences between occupational groups and clinical contexts. The large variation in physical activity and work demands indicates that future interventions should be tailored to specific occupational tasks within clinical contexts. Strategies such as workload redistribution to reduce prolonged or heavy physical and mental workloads or scheduled sitting breaks in standing-intensive roles may be relevant for workers with severe pain, but their effectiveness and feasibility should be evaluated carefully.

### Strengths and limitations

The use of device-based measurements of physical activity applying a machine learning model with high accuracy [24] provides valid estimates of physical activity and sedentary behavior [44]. Nonetheless, the methodology does not capture detailed work-related activities such as trunk flexion, arm elevation, or frequency of lifting, which are identified risk factors for MSP [12, 45]. Furthermore, self-perceived work demands were evaluated using NASA-TLX which is validated in employees in the healthcare sector [46]. However, the Norwegian translation and modified version used in the STUNTH study has not been formally validated. The original tool was designed to measure perceived workload related to a specific task (e.g., performing surgery or patient monitoring), while in our study it was applied to more broadly measure overall perceived work demand for an entire work shift. This may affect precision and interpretability and limit the comparison between other studies that have used NASA-TLX to investigate work demands.

Even though the subjective assessments of self-perceived work demands are complemented by physical activity measurements, the current design does not allow us to determine which specific activities or characteristics of the work shift were perceived as (physically) demanding or why. The physical activity measurements are also limited to time spent in different physical activity types which may limit interpretability across individuals with varying physical capacities, where the relative load could be more informative. However, this would require measures of physical capacity that were not available in our study population.

Physical behavior is compositional by nature, and evidence suggests that this should be considered when addressing causal questions [47]. However, given the descriptive aim of this study, and in line with guidelines for descriptive research that recommend careful consideration of covariate adjustment [32], we chose not to apply a conventional data analysis approach. Future studies addressing causal relations between physical activity, work demands and pain should use statistical methods that account for the interdependence of the different physical behaviors, such as compositional data analysis.

Data collection took place during the COVID-19 pandemic, which likely led to underrepresentation of staff from the busiest hospital clinics due to time constraints, increased patient loads, and infection control measures. As a result, outcomes such as perceived work demands and time spent standing or moving may have been underestimated in certain clinical settings. Additionally, the study may be affected by the healthy worker effect, as only about 50% of STUNTH participants agreed to wear activity monitors. Those who participated may have been healthier or more physically active than non-participants, consistent with other Norwegian studies showing lower self-reported health and higher rates of non-communicable diseases among non-participants [48].

Finally, the diversity in our study population is both a strength and limitation. It expands the literature by adding a broader sample of hospital occupations not involved in direct patient care, such as white-collar workers and maintenance personnel. However, this heterogeneity warrants caution when comparing the results with previous studies focused on more homogenous occupational groups. Moreover, stratifying and presenting results by both occupation and clinical context further adds to existing literature as it shows that work demands and physical activity levels are context specific, as it varies not only by occupation, but also by which clinical setting one is employed. This hierarchical structure (individual participant nested within occupations, nested within clinical setting) also provides a descriptive reference for future studies that should account for these dependencies in their analyses.

## Conclusion

Hospital employees with long-lasting MSP reported higher perceived overall work demands and higher perceived physical demands, but no significant differences in proportion of time spent in device-measured physical activity types compared to employees without MSP.

## Supplementary Information

Additional file 1: Descriptive statistics of the included participants, sorted by occupational group and clinical contex. Table of descriptive statistics.

Additional file 2: Amount (%) of reported cases of musculoskeletal pain regions. Figure of musculoskeletal pain regions, sorted by most affected regions.

Additional file 3: Additional file 3 Median perceived scores for overall workload, and all sub-dimensions of NASA-TLX by occupation. Table of median scores for all sub-dimensions of work demands from the NASA-Task load index for all occupational groups.

Additional file 4: Median perceived scores for overall workload, and sub-dimensions of NASA-TLX according to musculoskeletal pain characteristics. Table of median scores sub-dimensions of work demands from the NASA-Task load index for all musculoskeletal pain categories.

## Data Availability

Due to participant confidentiality, participant data are not publicly available.

## Abbreviations

MSP: Musculoskeletal pain
NASA-TLX: NASA Task Load Index

## References

[1] Ferrari AJ, Santomauro DF, Aali A, Abate YH, Abbafati C, Abbastabar H et al. Global incidence, prevalence, years lived with disability (YLDs), disability-adjusted life-years (DALYs), and healthy life expectancy (HALE) for 371 diseases and injuries in 204 countries and territories and 811 subnational locations, 1990–2021: a systematic analysis for the Global Burden of Disease Study 2021. Lancet (London, England). 2024;403(10440):2133–61. 10.1016/S0140-6736(24)00757-8 PMC11122111 38642570

[2] Moberg L. Utviklingen i legemeldt sykefravær Året 2023. Oslo: Norwegian Labour and Welfare Administration; 2024.

[3] Bakke B, Degerud EMM, Gravseth HMU, Hanvold TN, Løvseth EK, Mjaaland B et al. Faktabok om arbeidsmiljø og helse 2021. Status og utviklingstrekk. STAMI-rapport. 2021.

[4] Davis KG, Kotowski SE. Prevalence of Musculoskeletal Disorders for Nurses in Hospitals, Long-Term Care Facilities, and Home Health Care: A Comprehensive Review. Hum Factors. 2015;57(5):754–92. 25899249 10.1177/0018720815581933

[5] Jacquier-Bret J, Gorce P. Prevalence of body area work-related musculoskeletal disorders among healthcare professionals: a systematic review. Int J Environ Res Public Health. 2023;20(1):841. 10.3390/ijerph20010841 PMC9819551 36613163

[6] Hämmig O. Work-and stress-related musculoskeletal and sleep disorders among health professionals: a cross-sectional study in a hospital setting in Switzerland. BMC Musculoskelet Disord. 2020;21(1):1–11. 10.1186/s12891-020-03327-w PMC7243303 32438929

[7] El-Tallawy SN, Nalamasu R, Salem GI, LeQuang JAK, Pergolizzi JV, Christo PJ. Management of Musculoskeletal Pain: An Update with Emphasis on Chronic Musculoskeletal Pain. Pain Ther. 2021;10(1):181–209. 33575952 10.1007/s40122-021-00235-2 PMC8119532

[8] Main CJ, de Williams C. Musculoskeletal pain. BMJ (Clinical Res ed). 2002;325(7363):534–7. 10.1136/bmj.325.7363.534 PMC1124059 12217996

[9] Lindegård A, Larsman P, Hadzibajramovic E, Ahlborg G. The influence of perceived stress and musculoskeletal pain on work performance and work ability in Swedish health care workers. Int Arch Occup Environ Health. 2014;87(4):373–9. 23609321 10.1007/s00420-013-0875-8 PMC3996278

[10] Helse-og omsorgsdepartementet. Meld. St. 7 (2019–2020). Nasjonal helse- og sykehusplan 2020-2023. 2022. Available from: https://www.regjeringen.no/no/dokumenter/meld.-st.-7-20192020/id2678667/.

[11] World Health Organization. Global strategy on human resources for health: Workforce 2030. 2016. Available from: https://iris.who.int/handle/10665/250368.

[12] Da Costa BR, Vieira ER. Risk factors for work-related musculoskeletal disorders: a systematic review of recent longitudinal studies. Am J Ind Med. 2010;53(3):285–323. 19753591 10.1002/ajim.20750

[13] European Agency for Safety and Health at Work (EU-OSHA). Summary - OSH in figures in the health and social care sector 2024. Available from: https://osha.europa.eu/en/publications/summary-osh-figures-health-and-social-care-sector.

[14] Madsen IE, Gupta N, Budtz-Jørgensen E, Bonde JP, Framke E, Flachs EM, et al. Physical work demands and psychosocial working conditions as predictors of musculoskeletal pain: a cohort study comparing self-reported and job exposure matrix measurements. Occup Environ Med. 2018;75(10):752–8. 30045952 10.1136/oemed-2018-105151 PMC6166595

[15] Christensen JO, Johansen S, Knardahl S. Psychological predictors of change in the number of musculoskeletal pain sites among Norwegian employees: a prospective study. BMC Musculoskelet Disord. 2017;18(1):140. 28376786 10.1186/s12891-017-1503-7 PMC5379631

[16] Gerr F, Fethke NB, Merlino L, Anton D, Rosecrance J, Jones MP, et al. A prospective study of musculoskeletal outcomes among manufacturing workers: I. Effects of physical risk factors. Hum Factors. 2014;56(1):112–30. 24669547 10.1177/0018720813491114

[17] Prince SA, Elliott CG, Scott K, Visintini S, Reed JL. Device-measured physical activity, sedentary behaviour and cardiometabolic health and fitness across occupational groups: a systematic review and meta-analysis. Int J Behav Nutr Phys Activity. 2019;16:1–15. 10.1186/s12966-019-0790-9 PMC6444868 30940176

[18] Skarpsno ES, Nilsen TIL, Sand T, Hagen K, Mork PJ. Physical work exposure, chronic musculoskeletal pain and risk of insomnia: longitudinal data from the HUNT study, Norway. Occup Environ Med. 2018;75(6):421–6. 29674486 10.1136/oemed-2018-105050

[19] Coenen P, Willenberg L, Parry S, Shi JW, Romero L, Blackwood DM, et al. Associations of occupational standing with musculoskeletal symptoms: a systematic review with meta-analysis. Br J Sports Med. 2018;52(3):176–83. 27884862 10.1136/bjsports-2016-096795

[20] Colpitts KN, Dias JLG, Faulkenberry TJ, Bozer ALH. Investigation of the relationship between perceived mental workload and chronic pain. Psi Chi J Psychol Res. 2023;28(4):247–55.

[21] de Vries HJ, Reneman MF, Groothoff JW, Geertzen JHB, Brouwer S. Self-reported Work Ability and Work Performance in Workers with Chronic Nonspecific Musculoskeletal Pain. J Occup Rehabil. 2013;23(1):1–10. 22661341 10.1007/s10926-012-9373-1 PMC3563949

[22] Geurts SA, Sonnentag S. Recovery as an explanatory mechanism in the relation between acute stress reactions and chronic health impairment. Scand J Work Environ Health. 2006;6:482–92. 10.5271/sjweh.1053 17173204

[23] Vandenbroucke JP, von Elm E, Altman DG, Gotzsche PC, Mulrow CD, Pocock SJ, et al. Strengthening the Reporting of Observational Studies in Epidemiology (STROBE): explanation and elaboration. Int J Surg. 2014;12(12):1500–24. 25046751 10.1016/j.ijsu.2014.07.014

[24] Bach K, Kongsvold A, Bårdstu H, Bardal EM, Kjærnli HS, Herland S, et al. A Machine Learning Classifier for Detection of Physical Activity Types and Postures During Free-Living. J Meas Phys Behav. 2022;5(1):24–31.

[25] Hart SG, Staveland LE. Development of NASA-TLX (Task Load Index): Results of Empirical and Theoretical Research. In: Hancock PA, Meshkati N, editors. Human Mental Workload. Advances in Psychology. North-Holland. 1988;52:139–83.

[26] Hart SG, editor. NASA-task load index (NASA-TLX); 20 years later. Proceedings of the human factors and ergonomics society annual meeting; 2006: Sage publications Sage CA: Los Angeles, CA.

[27] Grier RA, editor. How high is high? A meta-analysis of NASA-TLX global workload scores. Proceedings of the human factors and ergonomics society annual meeting; 2015: Sage Publications Sage CA: Los Angeles, CA.

[28] Fredheim OM, Borchgrevink PC, Landmark T, Schjodt B, Breivik H. [A new schedule for the inventory of pain]. Tidsskr Nor Laegeforen. 2008;128(18):2082–4. 18846126

[29] Cleeland CS, Ryan KM. Pain assessment: global use of the Brief Pain Inventory. Ann Acad Med Singap. 1994;23(2):129–38. 8080219

[30] Boonstra AM, Preuper HRS, Balk GA, Stewart RE. Cut-off points for mild, moderate, and severe pain on the visual analogue scale for pain in patients with chronic musculoskeletal pain. Pain^®^. 2014;155(12):2545–50. 25239073 10.1016/j.pain.2014.09.014

[31] Jelsness-Jørgensen L-P, Moum B, Grimstad T, Jahnsen J, Opheim R, Prytz Berset I, et al. Validity, reliability, and responsiveness of the brief pain inventory in inflammatory bowel disease. Can J Gastroenterol Hepatol. 2016;2016(1):5624261. 27446848 10.1155/2016/5624261 PMC4930809

[32] Lesko CR, Fox MP, Edwards JK. A Framework for Descriptive Epidemiology. Am J Epidemiol. 2022;191(12):2063–70. 35774001 10.1093/aje/kwac115 PMC10144679

[33] World Medical Association. World Medical Association Declaration of Helsinki: ethical principles for medical research involving human participants. JAMA. 2025;333(1):71–4. 39425955 10.1001/jama.2024.21972

[34] Kim SS, Okechukwu CA, Dennerlein JT, Boden LI, Hopcia K, Hashimoto DM, et al. Association between perceived inadequate staffing and musculoskeletal pain among hospital patient care workers. Int Arch Occup Environ Health. 2014;87(3):323–30. 23475312 10.1007/s00420-013-0864-y PMC5321209

[35] Gorce P, Jacquier-Bret J. Work-related musculoskeletal disorder prevalence by body area among nurses in Europe: systematic review and meta-analysis. J Funct Morphol Kinesiol. 2025;10(1):66. 10.3390/jfmk10010066 PMC11843921 39982306

[36] Matre D, Christensen JO, Mork PJ, Ferreira P, Sand T, Nilsen KB. Shift work, inflammation and musculoskeletal pain-The HUNT Study. Occup Med (Lond). 2021;71(9):422–7. 34551112 10.1093/occmed/kqab133

[37] Kinge JM, Knudsen AK, Skirbekk V, Vollset SE. Musculoskeletal disorders in Norway: prevalence of chronicity and use of primary and specialist health care services. BMC Musculoskelet Disord. 2015;16:75. 25887763 10.1186/s12891-015-0536-z PMC4392859

[38] Skovlund SV, Bláfoss R, Sundstrup E, Andersen LL. Association between physical work demands and work ability in workers with musculoskeletal pain: cross-sectional study. BMC Musculoskelet Disord. 2020;21(1):1–8. 10.1186/s12891-020-03191-8 PMC7071559 32171283

[39] Jakobsen MD, Sundstrup E, Brandt M, Jay K, Aagaard P, Andersen LL. Physical exercise at the workplace reduces perceived physical exertion during healthcare work: cluster randomized controlled trial. Scand J Public Health. 2015;43(7):713–20. 26156941 10.1177/1403494815590936

[40] Tjøsvoll SO, Wiggen Ø, Gonzalez V, Seeberg TM, Elez Redzovic S, Frostad Liaset I, et al. Assessment of Physical Work Demands of Home Care Workers in Norway: An Observational Study Using Wearable Sensor Technology. Annals Work Exposures Health. 2022;66(9):1187–98. 10.1093/annweh/wxac052 PMC9664225 35959647

[41] Lunde L-K, Koch M, Knardahl S, Veiersted KB. Associations of objectively measured sitting and standing with low-back pain intensity: a 6-month follow-up of construction and healthcare workers. Scand J Work Environ Health. 2017;3:269–78. 10.5271/sjweh.3628 28272649

[42] Korshøj M, Jørgensen MB, Hallman DM, Lagersted-Olsen J, Holtermann A, Gupta N. Prolonged sitting at work is associated with a favorable time course of low-back pain among blue-collar workers: a prospective study in the DPhacto cohort. Scand J Work Environ Health. 2018;10(5):530–8. 10.5271/sjweh.3726 29542805

[43] Hallman DM, Gupta N, Heiden M, Mathiassen SE, Korshøj M, Jørgensen MB, et al. Is prolonged sitting at work associated with the time course of neck–shoulder pain? A prospective study in Danish blue-collar workers. BMJ Open. 2016;6(11):e012689. 28186937 10.1136/bmjopen-2016-012689 PMC5128958

[44] Kongsvold A, Flaaten M, Logacjov A, Skarpsno ES, Bach K, Nilsen TIL, et al. Can the bias of self-reported sitting time be corrected? A statistical model validation study based on data from 23 993 adults in the Norwegian HUNT study. Int J Behav Nutr Phys Act. 2023;20(1):139. 38012746 10.1186/s12966-023-01541-y PMC10680356

[45] Lohne FK, Xu K, Fimland MS, Palarea-Albaladejo J, Redzovic S. Association between musculoskeletal pain and exposures to awkward postures during work: a compositional analysis approach. Annals Work Exposures Health. 2024;68(5):522–34. 10.1093/annweh/wxae027 PMC11285145 38603465

[46] Said S, Gozdzik M, Roche TR, Braun J, Rössler J, Kaserer A, et al. Validation of the Raw National Aeronautics and Space Administration Task Load Index (NASA-TLX) Questionnaire to Assess Perceived Workload in Patient Monitoring Tasks: Pooled Analysis Study Using Mixed Models. J Med Internet Res. 2020;22(9):e19472–e. 32780712 10.2196/19472 PMC7506540

[47] Gupta N, Rasmussen CL, Holtermann A, Mathiassen SE. Time-Based Data in Occupational Studies: The Whys, the Hows, and Some Remaining Challenges in Compositional Data Analysis (CoDA). Annals Work Exposures Health. 2020;64(8):778–85. 10.1093/annweh/wxaa056 PMC7544002 32607544

[48] Asvold BO, Langhammer A, Rehn TA, Kjelvik G, Grontvedt TV, Sorgjerd EP, et al. Cohort Profile Update: The HUNT Study, Norway. Int J Epidemiol. 2023;52(1):e80–91. 35578897 10.1093/ije/dyac095 PMC9908054

