# Supplementary material for: Work demands and physical activity in hospital employees with different degrees of musculoskeletal pain: descriptive data from the STUNTH study, Norway
Source: BMC Musculoskelet Disord. 2026 May 21;27:683. doi: 10.1186/s12891-026-09972-x (PMC13464351; doi:10.1186/s12891-026-09972-x)
Supplement: Supplementary file 1 [file 12891_2026_9972_MOESM1_ESM.docx]

Additional file 1 Descriptive statistics of the included participants, sorted by occupational group and clinical context

| Occupation | Age Mean (SD) | Female, % | Permanent  employment, % | Employment  fraction (100%), % |
| --- | --- | --- | --- | --- |
| Total  (N=1413) | 42.6 (12.4) | 83.9 | 79.1 | 72.8 |
| Nurses  (n=321) | 35.1 (12.2) | 92.2 | 74.5 | 52.8 |
| Specialist nurses (n= 277) | 47.1 (10.0) | 92.1 | 87.3 | 70.1 |
| Laboratory technician  (n =143) | 40.1 (11.9) | 90.2 | 75.5 | 82.8 |
| Senior doctors^a)^  (n=115) | 45.4 (9.8) | 58.3 | 73.9 | 75.5 |
| Administrative personnel  (n=103) | 49.7 (11.7) | 84.5 | 84.5 | 87.5 |
| Management (n=96) | 50.4 (9.5) | 69.8 | 91.7 | 100 |
| Other patient related^b)^  (n=88) | 43.4 (12.6) | 77.3 | 79.6 | 65.3 |
| Facility and support staff  (n=86) | 45.2 (12.5) | 70.9 | 90.7 | 76.3 |
| Imaging technicians  (n=58) | 41.7 (11.7) | 86.2 | 86.2 | 75.0 |
| Therapists^c)^ (n=48) | 41.9 (13.5) | 91.7 | 70.8 | 84.1 |
| Junior doctors (n=40) | 34.8 (3.5) | 70.0 | 22.5 | 83.8 |
| Psychologists (n=38) | 37.5 (9.1) | 89.5 | 71.1 | 78.8 |
| Clinical context | Age Mean (SD) | Female, % | Permanent  employment, % | Employment  fraction (100%), % |
| Total  (N=1,413) | 42.6 (12.4) | 83.9 | 79.1 | 72.8 |
| Internal medicine (n=349) | 35.1 (12.2) | 92.2 | 74.5 | 52.8 |
| Surgery (n=222) | 47.1 (10.0) | 92.1 | 87.3 | 70.1 |
| Anesthesiology (n=191) | 40.1 (11.9) | 90.2 | 75.5 | 82.8 |
| Laboratory medicine ^d)^ (n=156) | 45.4 (9.8) | 58.3 | 73.9 | 75.5 |
| Psychiatry (n=143) | 49.7 (11.7) | 84.5 | 84.5 | 87.5 |
| Other contexts^e)^  (n=108) | 50.4 (9.5) | 69.8 | 91.7 | 100 |
| Maintenance (n=94) | 43.4 (12.6) | 77.3 | 79.6 | 65.3 |
| Radiology n= (80) | 45.2 (12.5) | 70.9 | 90.7 | 76.3 |
| Prevention and rehabilitation (n=36) | 41.7 (11.7) | 86.2 | 86.2 | 75.0 |
| Central administration and management (n=34) | 41.9 (13.5) | 91.7 | 70.8 | 84.1 |
| a) In the Norwegian context, senior doctors have finished their specialization (consultants) and junior doctors are residents in different stages of training.  b) Including medical secretaries, healthcare assistant, social workers and pedagogues c) Including physiotherapist and occupational therapists  d) Including laboratory technicians and neurophysiology technicians e) Including acute medicine, gynecology & obstetrics, otorhinolaryngology | | | | |
