# Supplementary material for: Work demands and physical activity in hospital employees with different degrees of musculoskeletal pain: descriptive data from the STUNTH study, Norway
Source: BMC Musculoskelet Disord. 2026 May 21;27:683. doi: 10.1186/s12891-026-09972-x (PMC13464351; doi:10.1186/s12891-026-09972-x)
Supplement: Supplementary file 2 [file 12891_2026_9972_MOESM2_ESM.docx]

Additional file 2: Amount (%) of reported cases of musculoskeletal pain regions. Participants are counted multiple times if they report pain in several regions.
