# Supplementary material for: Work demands and physical activity in hospital employees with different degrees of musculoskeletal pain: descriptive data from the STUNTH study, Norway
Source: BMC Musculoskelet Disord. 2026 May 21;27:683. doi: 10.1186/s12891-026-09972-x (PMC13464351; doi:10.1186/s12891-026-09972-x)
Supplement: Supplementary file 3 [file 12891_2026_9972_MOESM3_ESM.docx]

Additional file 3 Median perceived scores for overall workload, and all sub-dimensions of NASA-TLX by occupation

|  | Total score |  | Mental demand |  | Physical Demands |  | Temporal Demands | |  | | Effort | |  | | Performance | |  | | Frustration | |  |
| --- | --- | --- | --- | --- | --- | --- | --- | --- | --- | --- | --- | --- | --- | --- | --- | --- | --- | --- | --- | --- | --- |
| Occupation | Median  (IQR) |  | Median  (IQR) |  | Median  (IQR) |  | Median  (IQR) | |  | | Median  (IQR) | |  | | Median  (IQR) | |  | | Median  (IQR) | |  |
| **Overall** (N=1,413) | 55.5 (45.2–64.9) |  | 72.6 (56.3–82.8) |  | 38.9 (13.7–65.3) |  | | 68.4  (50.5–81.8) | |  | | 70.0  (54.5–82.1) | |  | | 30.0  (15.2–82.1) | |  | | 51.1  (26.8–69.5) | |
| Nurses  (n=321) | 58.5  (44.2–67.0) |  | 70.5 (54.0–80.0) |  | 52.6 (27.4–72.1) |  | | 69.5  (48.0–83.2) | |  | | 68.7  (50.5–82.1) | |  | | 31.6  (16.8–60.0) | |  | | 51.6  (29.3–69.5) | |
| Specialist nurses (n= 277) | 57.1 (46.0–66.4) |  | 74.2 (58.6–82.6) |  | 50.5 (21.1–70.7) |  | | 66.3  (50.0–81.1) | |  | | 71.1  (56.8–84.2) | |  | | 24.7  (15.2–65.7) | |  | | 50.0  (23.7–71.6) | |
| Laboratory technician  (n =143) | 51.1 (43.4–60.0) |  | 72.6 (56.3–79.5) |  | 31.6 (13.6–56.3) |  | | 68.4  (50.0–81.8) | |  | | 67.9  (54.7–81.3) | |  | | 20.5  (9.1–54.7) | |  | | 44.2  (18.9–65.2) | |
| Senior doctors^a)^  (n=115) | 55.1 (47.1–60.7) |  | 77.9 (65.3–86.9) |  | 22.1 (10.0–45.3) |  | | 67.7  (55.3–77.9) | |  | | 68.4  (55.3–82.6) | |  | | 47.9  (17.2–67.4) | |  | | 49.5  (29.5–63.6) | |
| Administrative personnel  (n=103) | 50.8 (42.2–58.4) |  | 72.1 (60.0–80.8) |  | 10.1 (4.0–28.8) |  | | 66.3  (49.5–79.8) | |  | | 67.4  (52.6–78.9) | |  | | 26.8  (16.3–61.6) | |  | | 48.0  (26.8–69.5) | |
| Management (n=96) | 59.2 (49.8–65.5) |  | 78.4 (69.8–87.9) |  | 15.3 (7.1–37.6) |  | | 75.0  (59.5–87.1) | |  | | 76.0  (65.5–83.7) | |  | | 33.5  (19.1–67.1) | |  | | 55.1  (37.6–71.9) | |
| Other patient related^b)^  (n=88) | 53.2 (42.1–64.3) |  | 74.5 (55.5–84.2) |  | 21.3 (7.9–61.6) |  | | 65.2  (46.7-82.2) | |  | | 72.4  (52.6–86.1) | |  | | 25.1  (8.3–58.9) | |  | | 53.4  (30.8-71.9) | |
| Facility and support staff  (n=86) | 57.7 (44.0–66.7) |  | 51.3 (32.3–74.2) |  | 63.9 (48.9–80.0) |  | | 65.7  (51.1–80.0) | |  | | 65.3  (51.5–78.9) | |  | | 30.9  (10.5–60.0) | |  | | 52.6  (30.0–70.0) | |
| Imaging technician  (n=58) | 58.2 (51.0–71.0) |  | 69.2 (54.5–80.0) |  | 64.7 (46.3–78.4) |  | | 71.8  (52.6–84.2) | |  | | 71.6  (54.2–80.5) | |  | | 43.7  (11.1–47.7) | |  | | 58.5  (43.4–71.6) | |
| Therapists^c)^  (n=48) | 53.9 (45.4–64.8) |  | 73.4 (59.5–87.9) |  | 30.8 (13.9–64.4) |  | | 61.8  (52.6–82.5) | |  | | 67.8  (58.9–81.6) | |  | | 40.8  (20.3–60.6) | |  | | 50.5  (27.5–67.0) | |
| Junior doctors^a)^ (n=40) | 51.3 (41.8–63.3) |  | 70.8 (52.6–84.2) |  | 26.6 (9.8–44.5) |  | | 69.7  (47.9–81.2) | |  | | 72.2  (49.7–81.2) | |  | | 50.3  (22.6–65.7) | |  | | 36.3  (20.4–65.3) | |
| Psychologists (n=38) | 52.9 (45.1–60.4) |  | 81.7 (70.0–88.9) |  | 5.2 (2.6–17.4) |  | | 71.1  (49.5–85.3) | |  | | 73.2  (62.6–80.0) | |  | | 33.2  (21.6–55.3) | |  | | 55.0  (33.7–75.3) | |
| Abbreviations: NASA-TLX = NASA Task Load Index; IQR = Interquartile Range a) In the Norwegian context, senior doctors have finished their specialization (consultants) and junior doctors are residents in different stages of training.  b) Including medical secretaries, healthcare assistant, social workers and pedagogues c) Including physiotherapists and occupational therapists | | | | | | | | | | | | | | | | | | | | | |
