# Supplementary material for: Work demands and physical activity in hospital employees with different degrees of musculoskeletal pain: descriptive data from the STUNTH study, Norway
Source: BMC Musculoskelet Disord. 2026 May 21;27:683. doi: 10.1186/s12891-026-09972-x (PMC13464351; doi:10.1186/s12891-026-09972-x)
Supplement: Supplementary file 4 [file 12891_2026_9972_MOESM4_ESM.docx]

Additional file 4 Median perceived scores for overall workload, and sub-dimensions of NASA-TLX according to musculoskeletal pain characteristics

|  |  | Overall work demand | |  | Mental Demand | |  | Physical demand | |  | Temporal | |  | Effort | |  | Perform | |  | Frustration | | |
| --- | --- | --- | --- | --- | --- | --- | --- | --- | --- | --- | --- | --- | --- | --- | --- | --- | --- | --- | --- | --- | --- | --- |
| Pain variables | N | Med.  (IQR) | Median  diff.  (95% CI) |  | Med.  (IQR) | Median diff.  (95% CI) |  | Med.  (IQR) | Median dif.  (95% CI) |  | Med.  (IQR) | Median diff. (95% CI) |  | Med.  (IQR) | Median diff. (95% CI) |  | Med.  (IQR) | Median diff. (95% CI) |  | Med.  (IQR) | Median diff. (95% CI) | |
| **Overall** | 1413 | 55.5 (45.2–64.9) | N/A |  | 72.6 (56.3–82.8) | N/A |  | 38.9 (13.7–65.3) | N/A |  | 68.4 (50.5 – 81.8) | N/A |  | 70.0 (54.5 – 82.1) | N/A |  | 30.0 (15.2 – 82.1) | N/A |  | 51.1  (26.8 – 69.5) | N/A | |
| **MSP**  **last week** |  |  |  |  |  |  |  |  |  |  |  |  |  |  |  |  |  |  |  |  |  | |
| No | 316 | 51.8 (42.8–61.6) | Ref |  | 71.3 (53.5–81.1) | Ref |  | 30.3 (12.4–58.8) | Ref |  | 64.7 (47.7 – 78.9) | Ref |  | 67.9 (51.6 – 80.0) | Ref |  | 27.6 (14.9 – 61.6) | Ref |  | 48.4  (19.6 – 67.4) | Ref | |
| Short-term | 411 | 56.6 (44.4–64.7) | 2.3  (-0.3 to 4.9) |  | 70.5 (53.2–80.8) | -0.4  (-3.4 to 2.6) |  | 42.1 (14.1–66.3) | 8.1  (-1.0 to 17.4) |  | 67.4 (49.9 – 81.6) | 2.55  (-2.0 to 7.0) |  | 68.9 (53.7 – 81.1) | 1.7  (-1.9 to 5.3) |  | 27.4 (14.7 – 60.5) | -0.9  (-8.8 to 6.9) |  | 51.6  (26.8 – 69.5) | 2.9  (-1.9 to 7.6) | |
| Long-lasting | 574 | 58.1 (48.2–66.2) | 5.78 (3.4 to 8.2) |  | 74.2 (60.0–84.2) | 2.75  (-0.1 to 5.6) |  | 42.6 (14.1–68.4) | 10.3 (1.5 to 19.0) |  | 71.3 (52.6 – 83.2) | 5.9  (1.7 to 10.2) |  | 71.6 (56.8 – 83.2) | 3.78 (0.4 to 7.2) |  | 32.2 (15.8 – 63.2) | 4.3  (-3.1 to 11.6) |  | 52.6  (30.5 – 73.2) | 3.6  (-0.9 to 8.0) | |
| **Pain intensity** |  |  |  |  |  |  |  |  |  |  |  |  |  |  |  |  |  |  |  |  |  | |
| Continuous (NRS 0-10) | 983 | 56.6 (46.0–65.5) | 1.3  (0.6 to 2.0) |  | 72.6 (56.8–83.2) | 0.1  (-0.7 to 1.0) |  | 42.6 (14.2–66.8) | 5.3  (2.7 to 7.9) |  | 69.5 (51.1 82.8) | 1.3  (0.1 to 2.6) |  | 70.5 (54.7 – 82.1) | 1.2 (0.2 to 2.3) |  | 30.5 (15.3 – 62.6) | -0.6  (-2.9 to 1.8) |  | 52.5  (30.0 – 71.6) | 1.3 (0.1 to 2.6) | |
| No pain (NRS 0) | 36 | 51.2 (42.1–62.1) | Ref |  | 71.6 (60.6–78.9) | Ref |  | 44.7 (7.9–58.9) | Ref |  | 65.8 (38.1 – 78.2) | Ref |  | 65.8 (49.7 – 75.3) | Ref |  | 35.8 (13.7 – 55.6) | Ref |  | 49.5  (30.3 – 63.2) | Ref | |
| Mild Pain (NRS 1-3) | 741 | 56.4 (46.1–65.3) | 5.3  (-0.5 to 11.0) |  | 72.7 (56.8–83.3) | 3.6  (-2.7 to 9.9) |  | 40.0 (13.7–66.3) | 12.3  (-8.9 to 33.5) |  | 69.5 (51.1 – 82.3) | 3.4  (-6.8 to 13.7) |  | 70.0 (56.1 – 81.8) | 3.7 (-4.4 to 11.8) |  | 31.3 (16.2 – 62.6) | -3.8 (-22.0 to 14.4) |  | 51.6  (30.0 – 70.7) | 2.4  (-7.9 to 12.8) | |
| Moderate pain  (NRS 4-6) | 193 | 58.6 (47.1–66.6) | 7.5  (1.4 to 13.7) |  | 72.2 (57.1–83.2) | 1.6  (-5.1 to 8.4) |  | 50.3 (17.4–71.6) | 25.0 (2.3 to 47.6) |  | 70.7 (54.2 – 84.7) | -4.6  (-6.3 to 15.5) |  | 73.7 (54.7 – 85.3) | 6.2 (-2.4 to 14.9) |  | 28.3 (13.6 – 63.2) | -7.3  (-26.7 to 12.1 |  | 55.6  (30.5 – 75.8) | 6.2 (-4.8 to 17.2) | |
| Severe pain (NRS 7-10) | 13 | 60.5 (51.0–68.5) | 9.4  (-1.5 to 20.4) |  | 71.1 (33.7–81.6) | 1.05  (-10.9 to 13.1) |  | 53.1 (21.1–83.7) | 30.2  (-10.0 to 70.4) |  | 76.8 (51.6 – 87.4) | 10.9  (-8.5 to 30.3) |  | 80.0 (51.1 – 90.5) | 11.8 (-3.5 to 27.3) |  | 11.6 (8.9 – 52.1) | -20.7  (-55.2 to 13.8) |  | 57.9  (40.0 – 70.0) | 9.4 (-10.2 to 29.1) | |
| Abbreviations: IQR = interquartile range; CI = confidence interval; NRS = Numeric Rating Scale, 0-10 ^a^ Quantile regression analysis for median difference. Adjusted for age (continuous) and sex (male, female) | | | | | | | | | | | | | | | | | | | | | | |
